# CCR5 as a Coreceptor for Human Immunodeficiency Virus and Simian Immunodeficiency Viruses: A Prototypic Love-Hate Affair

**DOI:** 10.3389/fimmu.2022.835994

**Published:** 2022-01-27

**Authors:** Anna J. Jasinska, Ivona Pandrea, Cristian Apetrei

**Affiliations:** ^1^ Division of Infectious Diseases, Department of Medicine, School of Medicine, University of Pittsburgh, Pittsburgh, PA, United States; ^2^ Department of Molecular Genetics, Institute of Bioorganic Chemistry, Polish Academy of Sciences, Poznan, Poland; ^3^ Eye on Primates, Los Angeles, CA, United States; ^4^ Department of Pathology, School of Medicine, University of Pittsburgh, Pittsburgh, PA, United States; ^5^ Department of Infectious Diseases and Immunology, Graduate School of Public Health, University of Pittsburgh, Pittsburgh, PA, United States

**Keywords:** CCR5, human immunodeficiency virus, simian immunodeficiency virus, delta 32, red-capped mangabey, sooty mangabey, African green monkey, virus transmission

## Abstract

CCR5, a chemokine receptor central for orchestrating lymphocyte/cell migration to the sites of inflammation and to the immunosurveillance, is involved in the pathogenesis of a wide spectrum of health conditions, including inflammatory diseases, viral infections, cancers and autoimmune diseases. CCR5 is also the primary coreceptor for the human immunodeficiency viruses (HIVs), supporting its entry into CD4^+^ T lymphocytes upon transmission and in the early stages of infection in humans. A natural loss-of-function mutation CCR5-Δ32, preventing the mutated protein expression on the cell surface, renders homozygous carriers of the null allele resistant to HIV-1 infection. This phenomenon was leveraged in the development of therapies and cure strategies for AIDS. Meanwhile, over 40 African nonhuman primate species are long-term hosts of simian immunodeficiency virus (SIV), an ancestral family of viruses that give rise to the pandemic CCR5 (R5)-tropic HIV-1. Many natural hosts typically do not progress to immunodeficiency upon the SIV infection. They have developed various strategies to minimize the SIV-related pathogenesis and disease progression, including an array of mechanisms employing modulation of the CCR5 receptor activity: (i) deletion mutations abrogating the CCR5 surface expression and conferring resistance to infection in null homozygotes; (ii) downregulation of CCR5 expression on CD4^+^ T cells, particularly memory cells and cells at the mucosal sites, preventing SIV from infecting and killing cells important for the maintenance of immune homeostasis, (iii) delayed onset of CCR5 expression on the CD4^+^ T cells during ontogenetic development that protects the offspring from vertical transmission of the virus. These host adaptations, aimed at lowering the availability of target CCR5^+^ CD4^+^ T cells through CCR5 downregulation, were countered by SIV, which evolved to alter the entry coreceptor usage toward infecting different CD4^+^ T-cell subpopulations that support viral replication yet without disruption of host immune homeostasis. These natural strategies against SIV/HIV-1 infection, involving control of CCR5 function, inspired therapeutic approaches against HIV-1 disease, employing CCR5 coreceptor blocking as well as gene editing and silencing of CCR5. Given the pleiotropic role of CCR5 in health beyond immune disease, the precision as well as costs and benefits of such interventions needs to be carefully considered.

## CCR5 Chemokine Receptor

### Role and Function in the Organism

CCR5, a C-C chemokine receptor 5 (formerly known as CC-CKR-5 or CKR5), is primarily involved in immune surveillance, inflammatory response, tumor formation and metastasis (1–3), pathogenesis of inflammatory diseases (4–6), asthma (7, 8), and cancer (2, 3). It plays a key role in the recruitment of the immune cells to inflammation sites by directing immune cell migration (chemotaxis) along the chemokine gradient (9, 10). CCR5 regulates trafficking and effector functions of memory/effector T lymphocytes, macrophages, and immature dendritic cells (11). Beyond its direct involvement in mediation of the immune processes, it acts as a suppressor of learning, memories and synaptic connections in the brain (12).

#### CCR5 Receptor and Its Native Ligands

CCR5 is a seven-transmembrane, G protein-coupled receptor (GPCR), a member of the family of class A GCPRs. As a GPCR, CCR5 comprises of seven transmembrane α-helices, three extracellular loops, three intracellular loops, an amino-terminal domain and a carboxyl-terminal domain (13).

The natural ligands for CCR5 include chemokines (small chemoattractant cytokines) involved in innate immunity, which are natural suppressors of HIV-1 infection (14–17): macrophage inflammatory proteins CCL3 (MIP-1 α) and CCL4 (MIP-1 *β*), CCL5 (RANTES - regulated on activation, normal T-cell expressed and secreted) and CCL3L1, the most potent among the agonists of CCR5 and HIV-1-suppressant (18). CCL7 (MCP-3) is, on the other hand, the main antagonist ligand of the CCR5 receptor (19). Activation of the CCR5 receptor by its agonist ligands stimulates cell migration and mediates inflammatory responses.

#### CCR5 Receptor Lifecycle

CCR5 activation occurs upon binding its agonist ligands and leads to stimulation of pertussis toxin-sensitive heterotrimeric *αβγ* G protein by catalysing the exchange of GTP for GDP in the G*α* subunit that triggers intracellular pathways involved in chemotaxis and activation of leukocytes (20). Upon ligand binding, CCR5 receptor undergoes rapid phosphorylation in the carboxy-terminal region that promotes desensitization and internalization regulated by *β*-arrestin, an adaptor protein causing sequestration of the receptor to clathrin-coated pits. Upon clathrin-mediated endocytosis, CCR5 receptor moves to endosomes and Golgi network, and then is recycled back to plasma membrane (21–23). The conformation of the CCR5 receptor is dynamically impacted through this process and dependent on cellular localization (24).

### CCR5 Expression

#### Cell and Tissue Expression

CCR5 is expressed on a wide array of bone-marrow-derived cells, including lymphocytes, monocyte/macrophages, granulocytes, T cells, and specialized immune cells including natural killer (NK) cells and regulatory T (Treg) cells, located in primary and secondary lymphoid organs, including thymus and spleen, nonhematopoietic peripheral tissues, such as epithelium, endothelium, vascular smooth muscles, fibroblasts, and in central nervous system in neurons, astrocytes, microglia (15, 25–29). In the normal adult brain, CCR5 is highly expressed in microglia, yet it is undetectable in neurons (30).

#### CCR5 Expression in Relation to Inflammation

Increased levels of CCR5 expression on mononuclear cells is characteristic to chronically inflamed tissues, suggesting that CCR5^+^ cells are recruited to the inflammatory sites (25). In addition to the lymphoid tissues, CCR5 expression is induced in the cortical neurons and transiently lowered in microglia/macrophages in response to stroke (30).

### Alterations of CCR5 Expression Through Genetic Deficiencies

**Figure 1:**
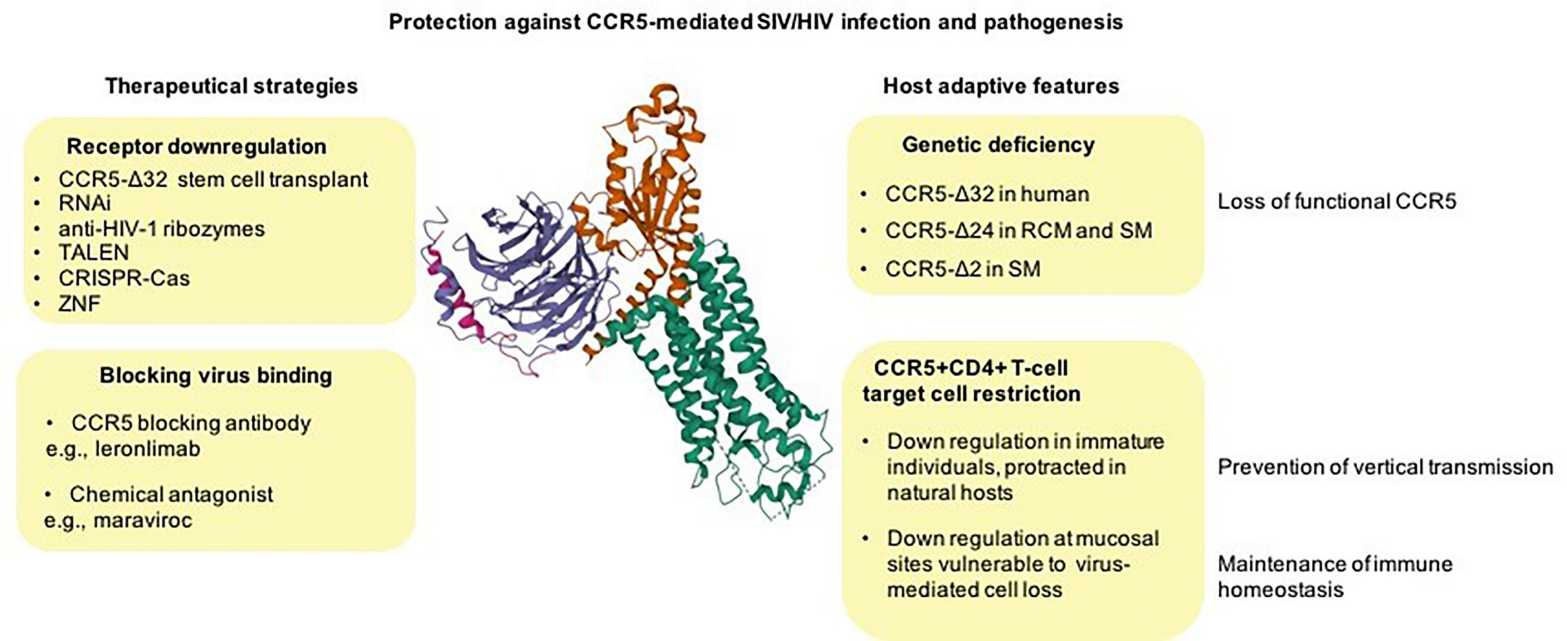
From natural control of CCR5 activity to therapeutic approaches against HIV disease. CCR5 null alleles, preventing CCR5-mediated virus entry, naturally emerged and raised to high frequency in different primate species most likely as a result of host adaptation to lethal pathogens (unknown ancient pathogen in humans and SIV in natural hosts) (top right). CCR5 downregulation of CCR5 on CD4+ T-cells may be an adaptive feature of natural hosts protecting against the vertical transmission of the virus (*via* breast feeding), and it may also represent an evolutionary adaptation to spare essential CD4+ T-cell subsets from killing by the virus (bottom right). Examples of approaches for therapeutic disruption of CCR5 expression include natural *CCR5* gene knockouts (resulting in functional cure), programmable nucleases, and gene silencing (top left) and approaches to blocking the virus fusion with cell membrane *via* chemical CCR5 receptor antagonists or antibodies (bottom left). Cryo-EM structure of the chemokine receptor CCR5 (green) in complex with RANTES and guanine nucleotide-binding protein Gi subunits alpha-1 (red), beta-1 (blue), and gamma-2 (magenta) was acquired from https://www.rcsb.org/3d-view/7F1R/1 wwPDB: Worldwide Protein Data Bank (13, 31, 32).

While CCR5 appears an essential player in various aspects of immune health, knockout alleles of this gene, leading to the loss of function of the CCR5 coreceptor, are present in different primate species at high frequency and with occurrence of null homozygous genotypes (**Figure 1**).

#### The CCR5-Δ32 Genetic Deficiency in Humans

CCR5 coreceptor expression on the cell surface can be prevented by a natural genetic variant, a 32-bp deletion (Δ32) observed in human populations. This mutation is localized in the region encoding the second extracellular loop of the receptor and results in a frameshift in the protein coding sequence leading to premature truncation of the normal CCR5 protein and abrogating its availability on the cell surface. The loss of function of the CCR5 gene modulates the risk for HIV transmission and counteract the pathogenesis of HIV infection (33–35).

#### Global Distribution of CCR5-Δ32 Allele in Human Populations

The CCR5-Δ32 allele is primarily observed in populations of European descent, where its average frequency is ~10%, while being virtually absent in SubSaharan Africans, Asians and Native Americans (34–37). Migrations have likely contributed to the global distribution of the CCR5-Δ32 allele, as, despite its predominant presence in Europe, high frequencies of CCR5-Δ32 were also observed in specific populations of European ancestry outside Europe, e.g., in South Africa (13%) and Chile (12%) (38). The CCR5-Δ32 is also present in African Americans, yet at low incidence (2%) (35) and in some Jewish populations, with the highest frequency in the Ashkenazis (11-20%) (39), where it probably emerged through admixture with people of European descent. The presence of this variant mainly in Euroasian populations suggests that the mutation occurred after their separation from the founders/ancestors of African, Asian, and Native American populations (40).

In Europe, the distribution of the CCR5-Δ32 variant shows marked clines, North-South and East-West. The highest frequency of the CCR5-Δ32 variant was observed among Northern Europeans, for example, in Finland and Russia (16%), Iceland (15%), Sweden (14%), Denmark (13%), Northern France (14%), and Norway (10%) (36, 37, 41, 42), while its lowest frequency was seen in Southern European and Mediterranean populations, such as Spain (7%), Italy (5.6%), Portugal (5.2%), Sardinia (4%), with the lowest prevalence being observed in Corsica (0.9%) (36, 37, 41).

#### Origins and Age of the Variant

The spatial distribution of the CCR5-Δ32 mutation, with the highest frequency in the Nordic countries suggests a Northern European origin of this variant (37, 41) and subsequent spreading out from the Scandinavian peninsula across Europe by the Vikings through their raids in the 8^th^-10^th^ centuries (41). While the role of the long-range dispersal consistent with the Viking mediated spread was demonstrated, other models raise the possibility that the allele arose outside of Scandinavia and moved into the region *via* dispersers from the South (43) or from the Finno-Ugrian tribes of Russia, where the mutation is frequent nowadays (37).

Several features of the CCR5-Δ32 mutation suggest that it had a unitary origin, subsequently becoming a subject to positive selection, as supported by: (a) its high frequency, (b) its virtual absence in other populations than those of European descent, (c) a striking gradient of this variant across Europe, as well as (d) a long-range disequilibrium in this CCR5 locus (36, 37, 44). Age estimates of the ancestral CCR5-Δ32 variant based on microsatellite analysis suggested that the deletion arose relatively recently, yet in a time frame that varies widely: about 700 years ago with an estimated range of 275-1,875 yrs (based on haplotype coalescence) (36); 3,400 years ago (based on the recombination frequency near the CCR5 locus); 1,400 years ago (based on the microsatellite mutation rate); or 2,000-2,200 years ago (based on mutation and crossover events) (37). Other studies suggested that the original single mutation occurred 2,500 years ago (44).

#### Neutral Drift vs. Selection in Favor of CCR5-Δ32

The relatively recent origin of the mutation, ranging between the Neolithic period (37) and Middle Ages (36), suggests that a positive selection or a selection acting on heterozygotes associated with selective advantage, rather than random drifts, could have driven the allele to the currently high frequency in the populations of European descent (37, 41). In comparison to the calculations based on the linkage disequilibrium, mutation rate, and the spatial allele distribution, which indicated the age of CCR5-Δ32 variant between several hundred to several thousand years, the age estimate for this variant was 127,500 years (i.e., two orders of magnitude higher), when its frequency was determined assuming neutral drift (36). This discrepancy points to intense natural selection, rather than random drift, as a force shaping the frequency of CCR5-Δ32 allele, suggesting an existence of local environmental factors, such as major pathogens, that have exerted a marked selective pressure on this locus in the historical time (43, 45).

#### Potential Selective Factors

Pathogens and infectious diseases represent major selective forces that shape the frequencies of alleles involved in protective immune mechanisms. Nowadays, the CCR5-Δ32 variant plays an important role against the HIV transmission in the human populations. An ancestor of HIV-1 originated from a cross-species transmission from chimpanzee to humans at the beginning of the 20^th^ century (46) and then it spread out in the European populations only in the 1980s (47, 48). Meanwhile, several cross-species transmissions of the SIVsmm that naturally infect the sooty mangabeys are at the origin of HIV-2 (49–52). These cross-species transmissions also occurred during the 20^th^ century (53). Since HIVs were passed to humans only recently, they have not had sufficient time to exert such profound selective effect on the allele frequency. Instead, several other pathogens and resultant diseases were proposed to drive the CCR5-Δ32 mutation to the contemporarily high frequencies.

The first proposed selective factor was the plague, which had a high mortality rate and was confined to Europe, where it persisted for ~300 years, from 1347 to 1670 (36). The plague hypothesis, which still remains one of the most “popular” concepts, despite some contradicting observations, proposed that macrophages infected with *Yersinia pestis* (54) were introduced in the human bloodstream by bites by fleas travelling on black rats in the Middle Ages (36), spreading the disease that killed ~40% of the population of Europe during epidemics, such as the Black Death of 1348–1350. *In vitro* studies showed, however, that although the *Ccr5*-deficient macrophages have a drastically reduced uptake of *Yersinia pestis* (an isolate from a fatal human case of plague), they experience a similar mortality with the wild-type *Ccr5*-expressing macrophages (55), suggesting that, if this model is representative to humans, the CCR5 deficiency did not have a protective effect against plague in people (56). The plague hypothesis was also challenged by the observation that plague may have not generated sufficient selective pressure for increasing the CCR5-Δ32 allele to contemporary frequencies (57). Instead, the selective raise of the CCR5-Δ32 allele was proposed to be attributed to smallpox *(Variola major)* caused by the poxvirus, based on population genetic analysis, which considered the temporal pattern and age-dependent nature of the diseases (57). The smallpox hypothesis, on the other hand, was opposed by the argument that the lethal form of smallpox emerged only recently (in England ~1628), not long before the introduction of variolation ~1750 and vaccination ~1800 (44) that gave the pathogen a narrow time window to push the mutant allele frequency to the current level (less than estimated 600 years needed for that) (57). As a selecting factor, other models considered recurrent epidemics of viral hemorrhagic fevers (“hemorrhagic plague”) affecting the eastern Mediterranean region since at least 1500 BC (44) or a pathogen spread during the time of the expansion of Roman Empire (58). A potential influence of other microbes, such as *Shigella*, *Salmonella*, and *Mycobacterium tuberculosis*, on the frequency of CCR5-Δ32 variant was also proposed (36).

Sabetti et al. reported that the variation of the CCR5-Δ32 was consistent with the pattern of neutral selection and estimated that the ancestral haplotype carrying CCR5-Δ32 variant might have arisen more than 5,000 years ago, with a certain probability that some selection has occurred thereafter (40).

### Common CCR5 Deficiencies in Nonhuman Primates (NHPs) That Are Natural Hosts of SIV

The case of CCR5-Δ32 allele in humans resembles deletions in the CCR5 gene present in the African monkeys of the genus *Cercocebus*, preventing CCR5 coreceptor-mediated SIV entry.

#### CCR5-Δ24 Mutation in Red-Capped Mangabey (RCM, *Cercocebus torquatus*) and Sooty Mangabey (SM, *C. atys*)

West African natural host species of SIVs, particularly the *Cercocebus* species, such as the RCM and the SM often carry a 24-bp deletion mutation in the *CCR5* gene (CCR5-Δ24) that causes an in-frame deletion of eight aminoacids in the fourth transmembrane region, abrogating the cell surface expression and coreceptor function of CCR5 for SIV entry (59, 60). The CCR5-Δ24 mutation in RCMs has a high frequency (86.6%) (60), exceeding that of the CCR5-Δ32 allelic variant in the human populations (44). It was observed in geographically distant RCM populations, from Gabon and Nigeria (and mangabeys in the US zoos), which demonstrates that its frequency is not due to local founder effects, but rather attributable to an old age and selective advantage of the variant (60, 61). The CCR5-Δ24 allele was also observed in SMs, yet at lower frequencies (4.1%) (60). The geographic ranges of RCMs and SMs are not overlapping at present days, as RCMs inhabit the swamps, mangroves and riverine forests along the Gulf of Guinea shore of Nigeria, Cameroon, Equatorial Guinea, Gabon, and the Gabon-Congo border (62), while the habitat of the SMs ranges in the forests at the Atlantic coast from Senegal to the Ivory Coast (63). The exact time of origin of the CCR5-Δ24 mutation in these two *Cercocebus* species is not known. The timing of divergence for these species is estimated at 2.29 MYA (64), suggesting either an ancient age of mutation before the split of these species, or its emergence following recurrent events. Unlike HIVs, which on the pandemic scale have been in the human populations for ~60 years, SIVs have been present in African NHPs for a much longer evolutionary time scale (65, 66) and potentially could have been a selective factor behind the high frequency of null CCR5 alleles. However, this hypothesis has yet to be confirmed.

#### CCR5-Δ2 Mutation in SM

In addition to the CCR5-Δ24 allele (that they carry at a frequency of 3%), SMs are also frequently (26%) carrying a 2-bp deletion in the *CCR5* gene (CCR5-Δ2), which, like the CCR5-Δ24 allele, encodes a truncated molecule that is not expressed at the cell surface (59).

The presence of these common deletion alleles of CCR5 in different primate species (CCR5-Δ32 in humans, CCR5-Δ24 in RCMs and SM, and CCR5-Δ2 in SM) suggests that the emergence and high frequencies of these alleles may represent a convergent evolution, yet it remains unclear what pathogens were driving these adaptations, most likely different for humans and African NHPs.

## CCR5 Role in HIV/SIV Infection

To infect CD4^+^ T cells in humans, HIV-1 utilizes CCR5 (mediating entry of R5 viruses) or CXCR4 (mediating entry of X4 viruses), or both entry coreceptors (67). CCR5 coreceptor is mostly expressed on memory CD4^+^ T cells, while CXCR4 is expressed on both memory and naive cells. The change in coreceptor usage towards CXCR4-tropism during the later stage of HIV-1 infection may contribute to accelerated disease progression (68–70). Meanwhile, in addition to CCR-5, HIV-2 uses GPR15 (BOB) and CXCR6 (BONZO) (71).

CCR5 density on the surface of CD4^+^ T cells is a key regulator of cell infectability and virus production, and a critical determinant of the HIV-1 disease progression (72). The extent of cell death correlates with the virus replication, and the capacity of HIV to induce cell death depends on the level of CCR5 expression on the surface of the CD4^+^ T cells (73). The density of CCR5 receptors on target cells is logarithmically correlated with HIV-1 viremia (72) and disease progression (74). *In vitro* studies suggested a dual role of CCR5 in determining HIV-1 production: as an entry coreceptor, it acts as a critical factor for infection, yet exerts only a moderate influence on the magnitude of viral loads, while as a postentry regulator of the HIV-1 life cycle, particularly at reverse transcription stage, it accounts for the logarithmic relation between the viremia and CCR5 density (75).

### HIV Binding

HIV entry *via* CCR5 receptor occurs through a series of processes, depending on the conformational state of both viral envelope protein and cellular receptor (24). CCR5 stabilizes the CD4-induced conformation of Env protein and anchors the virus near the cell surface (76). Chemokines that are native CCR5 ligands naturally restrict HIV-1 infection sterically, by masking the viral envelope glycoprotein gp120 binding sites and promoting CCR5 endocytosis, reducing the CCR5 cell surface level (77, 78). The second extracellular loop and amino-terminal domain of CCR5 receptor are critical for interacting with HIV Env protein and binding natural chemokine ligands, such as CCL4 and RANTES. While these molecules bind different regions, they both compete with the virus for the binding site (79, 80).

Biological activity of CCR5 depends on its conformations (81), which influences interaction with HIV gp120 and native chemokines (82). Some receptors have low binding affinity for native CCR5 chemokines and therefore chemokines are weak inducers of CCR5 endocytosis (82).

### CCR5-Δ32 Genetic Variant Has a Protective Action Against HIV Infection

The CCR5-Δ32 variant generates a nonfunctional entry coreceptor for HIV that does not support fusion between the virus and the target-cell membrane, thus preventing infection and pathogenesis. The homozygous CCR5-Δ32bp genotype (Δ32/Δ32) carriers (about 1% of Europeans) are highly protected from HIV-1 infection (33–35, 83–85), yet this protection is not complete, as rare cases of HIV infection were reported in the homozygotes (86–88). The Δ32bp knock-out of the *CCR5* gene was observed in cohorts of multiple HIV-exposed seronegative (HESN) individuals (33, 35), and resistance of circulating cells to HIV infection *in vitro* was reported (34).

The WT/Δ32 heterozygotes exhibited a reduced ability to support HIV-1 replication compared to the wild type homozygotes (WT/WT) (33–35), had reduced viral loads (33, 34, 36, 84), a slower rate of CD4^+^ T-cell depletion (84), resulting in a 2-3 years delayed progression to AIDS (33–35, 84, 85, 89), and improved virological response to antiretroviral therapy (90). CCR5-Δ32bp heterozygosity also appeared to be associated with reduced susceptibility to HIV-1 infection (91), yet this observation was not universally confirmed (92). The CCR5 WT/Δ32 genotype was also associated with protection from AIDS-related lymphoma, a non-Hodgkin’s B cell malignancy that is common in patients with AIDS (93, 94).

An increased prevalence of heterozygotes for the CCR5-Δ32 mutation was found in some, but not all, cohorts of HIV long-term nonprogressors (LTNP, i.e., HIV infected individuals with little or no clinical signs of progression), but it does not appear either essential or sufficient for protection against disease progression (87, 95). In elite controllers (ECs, which spontaneously control HIV replication to undetectable viral loads and maintain stable CD4^+^ T-cell counts), the prevalence of CCR5-Δ32 heterozygotes appears somewhat elevated compared to the general population, yet this difference is not striking (96).

#### Molecular Mechanism of Protection Conferred by the CCR5-Δ32 Allele

When compared to homozygotes with both normal copies of CCR5 WT/WT, CCR5 heterozygotes WT/Δ32 associate a >50% reduction in cell surface expression of CCR5, and display a lower infectability of blood cells by the M-tropic HIV-1 *in vitro* (97, 98). The abrogation of cell surface expression of CCR5 coreceptor is caused by the interruption of CCR5 transport to the cell membrane. While normal CCR5 protein can undergo both post-translational phosphorylation and/or cotranslational multimerization, the mutant CCR5-Δ32 can only form multimers and is incapable of being phosphorylated. In the CCR5 heterozygotes, the heterodimers between the normal CCR5 and mutant CCR5-Δ32 proteins are retained in the endoplasmic reticulum causing reduced cell surface expression of the functional CCR5 coreceptor (98).

#### Genetic Variation in CCR5 and Its Ligands May Influence CCR5 Functionality

The delayed progression to HIV disease was associated with other types of genetic variation in the *CCR5* locus. For example, variants located within the CCR5 promoter (99–102) showed regulation of CCR5 cell surface expression and of CD4^+^ T-cell apoptosis, as well as a correlation with HIV disease progression (103). The CCR5 promoter variant 59029 G/A reduced the activity of the CCR5 promoter by ~45% and resulted in ~4 years delayed progression to AIDS in the carriers of this mutation (104). Downregulation of active transcription of CCR5, paralleled with reduced cell surface expression of CCR5, was observed in a subset of elite and viremic controllers with an R5-resistance phenotype (105). The transcriptomic downregulation of CCR5 (9-fold) was associated with downregulation of multiple genes, including CCR2, in the 500 kb block around the CCR2-CCR5 locus on the chromosome 3p21 (105).

Also, genetic variation in genes coding ligands of CCR5 may influence the functionality of this receptor. For example, CCL3L, a HIV-1 suppressive chemokine, shows a copy number variation that is associated in a dosage dependent manner with susceptibility to HIV infection; lower number of copies of CCL3L are associated with an increased risk of HIV (106).

The effect of the CCR5 deficiency (Δ32/Δ32) in conferring nearly complete prevention of HIV-1 infection, was achieved through experimental manipulation that blocked HIV-1 entry into cells with an anti-CCR5 reagent (97). Given that the genetic variation lowering the CCR5 expression has an advantageous effect on taming HIV pathogenesis and did not seem to be associated with a deleterious phenotype in humans (33), interventions blocking or reducing the CCR5 expression emerged as promising approaches to the prevention and treatment of the HIV disease (107–115). However, while the initial studies suggested that the loss of function due to the CCR5-Δ32 does not bear marked impact on health, there is increasing evidence that CCR5 plays a complex role in organism homeostasis, and is not completely dispensable (45, 116–118).

### CCR5 Role in Infectious Diseases

Beyond SIV/HIV infection, CCR5 plays multiple roles in viral diseases (119), bacterial and parasitic infections (120). It is anticipated that CCR5-deficiency may exert several different, some mutually opposing, effects: (a) prevent infection with CCR5-tropic pathogens, (b) weaken the immune response to some pathogens, leading to increased susceptibility to infection, and (c) reduce CCR5-mediated inflammation, which can either hamper protective inflammatory response, or reduce problems related to excessive inflammation.

CCR5 is a key protective factor against some pathogens. For example, it promotes survival during infection with the West Nile virus (WNV), which can cause fatal encephalitis, by promoting leukocyte trafficking to the brain during the infection (116). However, genetic deficiency of CCR5 (Δ32/Δ32) confers a strong risk of symptomatic WNV infection associated with a fatal outcome (117, 121). In influenza patients, CCR5 deficiency causes a four-fold increased mortality (122). These findings warrant a question regarding the safety of some HIV therapies employing null CCR5 alleles and motivates a development of strategies blocking virus binding CCR5 while preserving the functionality of CCR5 as a chemokine receptor (118).

On the other hand, CCR5 is implicated in infections with CCR5-tropic pathogens, such as Dengue virus (123) and *Staphylococcus aureus* (124). CCR5-Δ32 mutation showed a protective effect against community acquired pneumonia caused by *Streptococcus pneumoniae* (125) and against a severe form of COVID-19 (126).

The CCR5-Δ32 variant plays a complex pro- and antimicrobial role in *Mycoplasma pneumoniae* infection, showing an association with development of chronic infection, yet also with a reduced risk of asthma development in infected children, when compared to children with a nondeleted version of CCR5 (127). Analogically, CCR5 null allele plays a protective effect against toxoplasmosis (*Toxoplasma gondii*) infection (128, 129), while the functional CCR5 receptor is an essential regulator of the inflammatory response following this parasitic infection (10, 130).

### Role of CCR5 Beyond Response to Microbes

CCR5-Δ32 mutation was implicated as a factor modulating the risk of neurodegenerative dementias (131, 132) and recovery after stroke and traumatic brain injury (30). CCR5 link with Alzheimer disease was suggested by several studies, yet contradicting association results were reported in the others (131). In the human populations, the CCR5-Δ32 allele was not significantly associated with neurodegenerative dementias, however, an earlier age of onset of neurodegenerative disease was observed in carriers of the CCR5-Δ32 allele, suggesting that the deletion may have a detrimental effect in the context of neurodegeneration (132). Humans that carry CCR5-Δ32 have better outcomes after stroke, with an enhanced motor recovery and reduced cognitive deficits (30). Based on that observation, CCR5 was proposed as “a translational target for neural repair in stroke and traumatic brain injury” (30). This is consistent with the observation in a mouse model that inhibition of CCR5 signaling enhanced neuroplasticity processes, learning and memory, while overexpression of CCR5 led to learning and memory deficits (12).

### A Nonprogressing SIV Infection in Natural Hosts

Many African NHP species (e.g., SM, RCM, African green monkey-AGM, mandrill-MND) carry species-specific SIVs, a family of viruses from which HIV evolved. Yet, in contrast to the progressing hosts, such as humans and macaques, the African species do not typically develop immunodeficiency despite many years of infection and high levels of viral replication (133–135).

#### Host-Pathogen Coevolution

This lack of disease progression in the natural hosts is attributed to the long-term host-pathogen coevolution spanning between hundreds of thousands to possibly millions of years (65, 66), which allowed for the development of protective mechanisms, including lower levels of immune activation upon infection (136–142). Such nonpathogenic SIV infections in natural hosts, some of which utilize specific CCR5 regulations to minimize pathogenesis, provide an insight into adaptive mechanisms protective against the disease (143).

#### Differences in Infection Between Natural and Non-Natural Hosts

SIV infection in its respective natural hosts is usually nonprogressive and presents the following main features: a) only a transient depletion of peripheral CD4^+^ T cell, b) absence of intestinal dysfunction and its deleterious consequences, allowing the maintenance of integrity of gut barrier, and c) resolution of immune activation after acute infection. These features are in stark contrast to the pathogenic SIV/HIV infection in a non-natural host, which is characterized by a) progressive CD4^+^ T-cell loss, b) disruption of the intestinal barrier leading to severe gut dysfunction, and c) chronic inflammation and immune activation (144).

Despite the fact that progressing and nonprogressing hosts display stark differences in the course of infection and pathogenesis, they share several common features of the lentiviral infection, such as the high virus replication rates and fast turnover of infected cells (145–147). Natural SIV infections are therefore different from HIV-1 long-term nonprogressors and SIV-infected RMs, in which the deleterious impact of HIV/SIV infection is minimized through a control of viral replication. Instead, it resembles more to the viremic HIV controllers, a small fraction of HIV-infected individuals that control disease progression by keeping at bay chronic inflammation and T-cell immune activation, in the context of a very active viral replication (148).

#### Target Cell Availability Shapes Susceptibility to Infection and the Extent of SIV Pathogenesis

The SIV’s usage of CCR5 coreceptor to infect its target cells (149) renders the cells coexpressing CCR5 and CD4 (T-cells, the monocytes/macrophages, dendritic cells) the main targets for SIV/HIV infection.

##### Tissue Expression

The natural hosts of SIV, both uninfected and SIV-infected, are characterized by markedly lowered abundance of CCR5^+^ CD4^+^ T cells at the mucosal sites, as well as in peripheral blood, lymph nodes and in bone marrow compared to pathogenic hosts, human and macaques (134). In addition to the CD4^+^ T cells, CCR5 expression on monocytes is lower in the natural host than in humans and macaques, yet to a lesser extent (134). Intermediate levels of CCR5 expression on the CD4^+^ T cells were observed in the chimpanzee, a non-natural host, which acquired its species-specific SIVcpz more recently than the African monkey hosts. Still, chimpanzees were infected in the wild for considerably longer periods than humans and macaques. It was therefore postulated that chimpanzees did not have sufficient evolutionary time to adapt well to the virus and thus remain vulnerable to its pathogenic effects (149). Indeed, studies in wild chimpanzees reported that they can progress to AIDS-like disease and develop CD4^+^ T cell depletion, also their mortality rate was 10-16-fold increase compared to uninfected chimpanzees (150, 151).

In nonprogressing hosts, downregulation of CCR5 expression on CD4^+^ T cells is associated with lower levels of infection than in non-natural hosts (e.g., SMs vs RMs) (152). In SMs, CD4^+^ T cells, in particular central memory cells, did not upregulate CCR5 in response to *in vitro* stimulation, and the low CCR5 expression on central memory cells was associated with reduced susceptibility to infection (152). This specific regulation of CCR5 expression on different cell types may protect from SIV infection (and subsequent death) the CD4^+^ T cell subsets critical to a mild course of infection, while the virus replicates in less dispensable cells (152). It was postulated that long-standing selective pressure of SIV has led to the adaptive shift toward immune functions less dependent on the CD4^+^ CCR5^+^ T cells in natural hosts (134).

##### Age-Related Regulation of CCR5 Expression on T Cells

The CD4^+^ T cells expressing CCR5 on their surface are the main targets for HIV/SIV infection in both natural and non-natural hosts. The CCR5 surface expression shows a distinctive ontogenetic pattern characterized by an increase of CCR5 expression with the host maturation; as a result, availability of CCR5^+^ CD4^+^ target T cells increases with age. In general, the levels of target cells are very low in newborns compared to adults, in both natural hosts of SIV (i.e., AGMs) and in non-natural vulnerable hosts, i.e., macaques and humans (153–156). This pattern, however, markedly varies between progressing and nonprogressing hosts with respect to the timing. The target cell maturation is programmed distinctively among different primate species: (a) rapid in RMs, reaching CCR5^+^ CD4^+^ T-cell levels comparable to those in adults by the age of 9 months i.e., at the end of lactation (154, 155, 157); (b) intermediate in humans, with a gradual increase, reaching the adult level by 5-6 years i.e., long after weaning, yet still during childhood (158); (c) slow in natural hosts, in which at the end of the lactation period, the levels of target cells are not significantly different from newborns and much lower than in adults, and an increased CCR5 expression on CD4^+^ T cells only occurs at sexual maturity (144, 153, 159).

##### Delayed Maturation of Target Cells Protects Against Mother-to-Child Transmission (MTIT)

Vertical transmission of HIV and SIV can occur *in utero*, *intrapartum*, and postnatally, through breastfeeding (BFT) (160). Humans and NHPs show striking species-specific differences in the timing of maturation of the CCR5 expression on CD4^+^ T cells (i.e., onset of CD4^+^ CCR5^+^ T target cells) in immature individuals: early in RM, intermediate in humans, and later in AGMs. The increasing age of target cells availability was paralleled by the rates of BFT: 60% in RMs (161), 29% in humans (162), and 0-5% in AGMs (66, 153, 159) suggesting that the delayed maturation of the SIV target cells in natural hosts compared to pathogenic hosts may be the factor behind the lack of/low SIV BFT (144).

These observations are concordant with the age-related increase of SIV transmission in natural populations of AGMs in West Africa (sabaeus) and South Africa (pygerythrus/vervet) (66, 159) and ontogenetic changes in the abundance of CCR5^+^ CD4^+^ T cells in the blood of sabaeus AGMs. Among the uninfected monkeys, the levels of CCR5^+^ on circulating CD4^+^ T cells are low in infants and juveniles, and markedly increased in adults, that usually become infected at the age of sexual maturity (159). While there is no significant difference in the mean levels of target cells between SIV infected and uninfected adults, in immature individuals the availability of target cells is positively associated with the SIV infection status (159). A convergent observation was made in infants of rhesus macaques experimentally exposed to SIV treated with maraviroc - the maraviroc treatment prevented vertical SIV transmission only in individuals with naturally low levels of CCR5 on the CD4^+^ T cells prior to the treatment (163). The susceptibility to infection is proportional to the target cell availability at mucosal sites (164) and therefore the natural restriction of CCR5 expression in young individuals may represent a strategy to protect target cells from infection.

##### Restriction of CCR5 Expression and CD4^+^ CCR5^+^ T Cells at Mucosal Sites

In comparison to non-natural hosts, natural host show much lower expression of CCR5 on CD4^+^ T cells (but not CD8^+^ T cells) at mucosal sites, in particular in the gut, that leads to overall low numbers of CCR5^+^ CD4^+^ T target cell (134, 153), especially memory cells, at these locations (134). These adaptive roles of CCR5^+^ CD4^+^ target cell restriction suggest that interventions mimicking the natural phenomenon of limited abundance of target cells at critical developmental periods and tissues may protect against transmission and excessive cell death, respectively.

Despite the difference in availability of target cells supporting viral replication, both natural and non-natural hosts show high viremia. It brings up a question, if not CD4^+^ CCR5^+^ T cells, which other cells facilitate virus replication. A plausible explanation is that SIVs can infect cells in natural hosts through other coreceptors than CCR5, and in this way protect target cells and specific anatomical sites from SIV pathogenesis, and prevent vertical SIV transmission (153).

### SIV Usage of CCR5 and Alternative Coreceptors in Natural Hosts

SIVs predominantly use the canonical CCR5-mediated cell entry pathway (165), yet they can also use other coreceptors, usually in addition to CCR5: CXCR4, CXCR6 (AKA BONZO, STRL33), orphan receptors GPR1 and GPR15, as demonstrated through *in vitro* studies (165–169). A unique alternative coreceptor usage, CCR2, was developed by SIVrcm and SIVmm (59, 61, 170, 171).

#### SIVrcm Counters the CCR5 Deficiency by Altered Coreceptor Use

**Figure 2:**
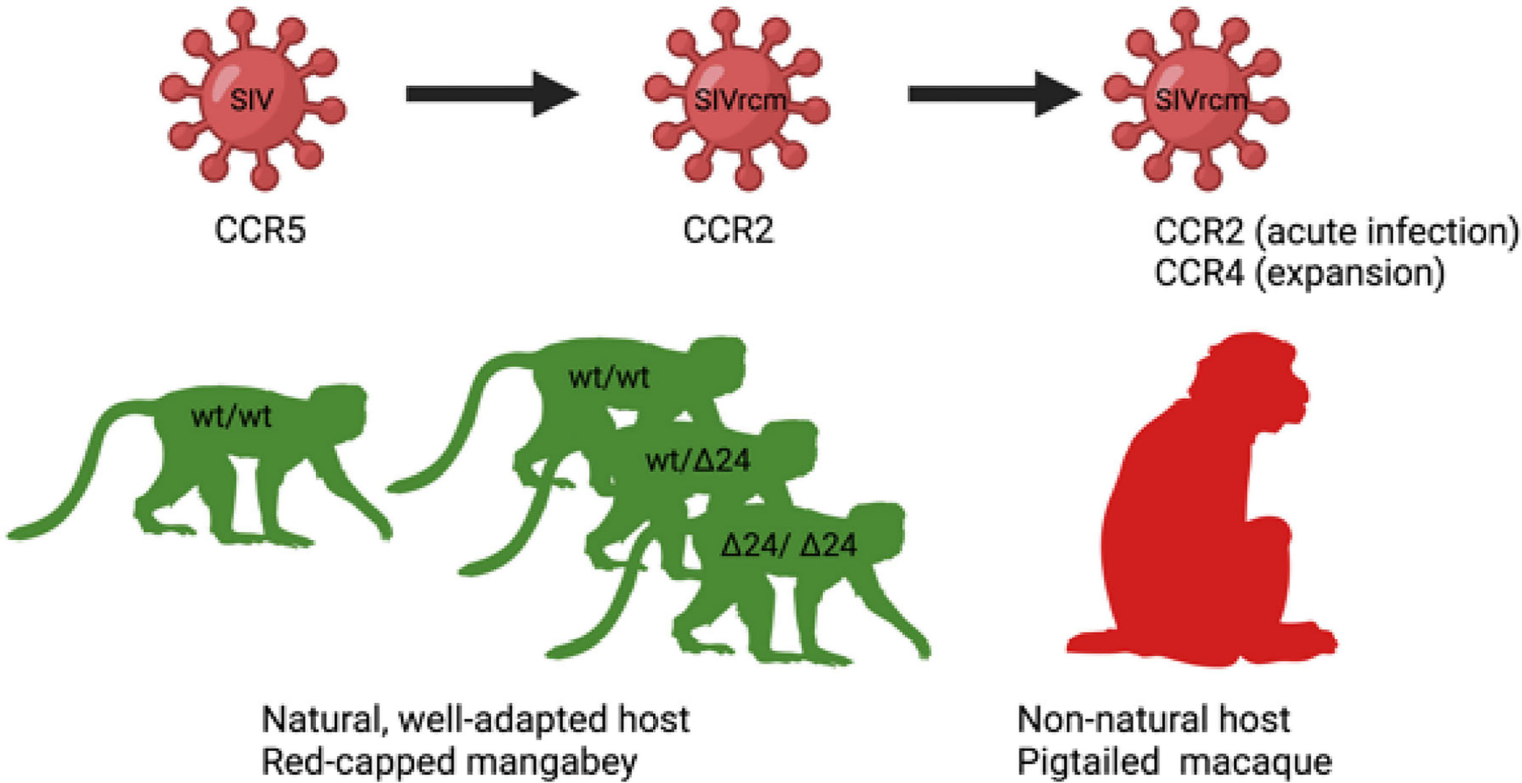
Host-pathogen co-adaptations in a natural (left) and non-natural host (right). On the left, a putative origin of a unique among SIVs CCR2-tropic SIVrcm infecting red-capped mangabey RCM: A high frequency of CCR5-Δ24 mutation, disrupting CCR5 function and protecting from infection with CCR5-tropic SIV, led to the virus adaptation *via* changing the usage of CCR5 to CCR2b for cell entry (60). On the right, SIVrcm adaptation of coreceptor usage to a new host: experimental SIVrcm infection of pigtailed macaque, a natural host, showed a CCR2 usage in early infection and expansion of coreceptor usage to CCR4 demonstrating that lentiviral adaptation may occur rapidly through strain selection (170).

As a result of the high frequency of deletion alleles, some RCMs and SMs are homozygous for nonfunctional CCR5 variants and therefore completely lack the functional CCR5 receptor on the cell surface. The frequency of null homozygotes in RCMs exceeds 70% (60). However, the CCR5 null genotype in RCMs and SMs is not sufficient to protect them from SIV infection *in vivo* (59–61, 172–174) in contrast to homozygotes of CCR5-Δ32 in humans that are nearly completely protected from HIV infection. This suggests that SIVs infecting these species bypassed the inactivated CCR5-mediated entry pathway by developing the ability to use alternative receptors (**Figure 2**).

##### SIVrcm Naturally Infecting RCMs Displays a Unique CCR2-Tropism

In spite of being frequently CCR5-Δ24 homozygous and thus widely protected against infection with a CCR5-tropic SIV, the RCMs are naturally infected with a species-specific SIVrcm. The SIVrcm strains collected from naturally-infected RCMs from distant geographic locations, Gabon and Nigeria, are members of the same lineage of SIV (60, 61). SIV prevalence in RCMs is significant, about 22% (175, 176). The explanation for this successful SIVrcm spread in the RCM populations is that the virus utilizes CCR2b as the main coreceptor for virus entry, unlike the vast majority of SIV and HIV strains that use CCR5 (60, 177). The high allelic frequency of the CCR5-Δ24 in RCMs paralleled with the unique among primate SIVs R2b-tropism of SIVrcm suggest that the CCR2 coreceptor usage may have been acquired by SIVrcm as an adaptation to CCR5 genetic defects in its host.

CCR2 is mainly expressed on monocytes (long lived cells) and nearly absent on T lymphocytes (short lived cells), and therefore it could be anticipated that, upon experimental infection, SIVrcm would show *in vivo* tropism toward monocytes (170). Yet, such an experimental infection of pigtailed macaques led to a surprising pattern of viral replication characteristic for short lived cells and a significant CD4^+^ T cell loss in the intestine and blood, in particular effector memory CD4^+^ T cells, and only a minimal monocyte depletion (170). These pathogenic features were explained by an *in vivo* expansion of the SIVrcm tropism upon infection of the pigtailed macaques upon expanded coreceptor use to CCR4. This coreceptor use expansion led to a selective depletion of CCR4-expressing memory CD4^+^ T cells (170). CCR4 was indeed reported to be expressed mainly on lymphocytes and only at very low levels on monocytes (178).

#### Alternative Pathways for CD4^+^ T Cell Entry: SIVsab in AGMs and SIVsmm in SM

Blocking of CCR5 coreceptor *in vitro* did not prevent SIV infection in circulating lymphocytes of SMs or AGMs (171) suggesting an existence of alternative entry pathways. In both cases, in addition to the CCR5 coreceptor, CXCR6 was an efficient entry pathway of SIV in *in vitro* experiments. Thus, SIVagm and SIVsmm utilize, in addition to CCR5, CXCR6 and GPR15 (171, 179). Additionally, SIVsmm utilizes GPR1 less frequently (59). *In vitro*, CCR5 appears nonessential for SIVsmm infection in SMs as SIVsmm glycoprotein can interact with GPR15 and CXCR6 supporting a similar level of infection as that mediated *via* CCR5 (59).

Alternative pathways are exclusively responsible for SIVsmm replication in animals that genetically lack functional CCR5, while both CCR5 and alternative coreceptors may be used in hosts where both CCR5 and alternative pathways are available (180). It was postulated that the usage of alternative non-CCR5-mediated pathways in natural hosts may be a counter measure to minimize pathogenicity of infection, yet still maintain high virus replication levels by directing the virus to different cell subsets, less critical to the maintenance of immune homeostasis (59).

Note, however, that these *in vitro* experiments were carried out using SIV clones, some of them derived after passage through pathogenic hosts, which might have impacted their *in vitro* tropism. *In vivo*, SIVsab was shown to preferentially infect and deplete central and effector memory cells (181), being thus possible that, *in vivo*, the transmitted-founder viruses preferentially infect CCR5-expressing CD4^+^ T cells, thus recapitulating the pathogenesis of HIV-1 transmission, when transmitted founder HIV strains infect exclusively CCR5-expressing cells and display specific phenotypes (182–184). It is very likely that, as infection progresses, SIVs confronted with a low availability of the CCR5^+^-expressing cells, expand their *in vivo* tropism towards the other coreceptors described above (59, 171, 179, 180).

This idea is also supported by reports of coreceptor expansion for SIVsmm to efficiently infect naïve cells. While typically SM do not experience chronic CD4^+^ T-cell loss or clinical signs of disease, a small subset of SMs showed a profound CD4^+^ T-cell depletion associated with carrying SIVsmm variants with an expanded use of SM-derived coreceptos (180) and human coreceptors, including CXCR4 (180, 185, 186), and generally expanded tropism. These coreceptors may support virus replication in SMs that have restricted CCR5 expression and lack functional CCR5 due to loss of function mutations.

#### Loss of Ability to Use CXCR6 and Switch Towards Virtually Exclusive Use of CCR5 by Pathogenic Lentiviruses

**Figure 3:**
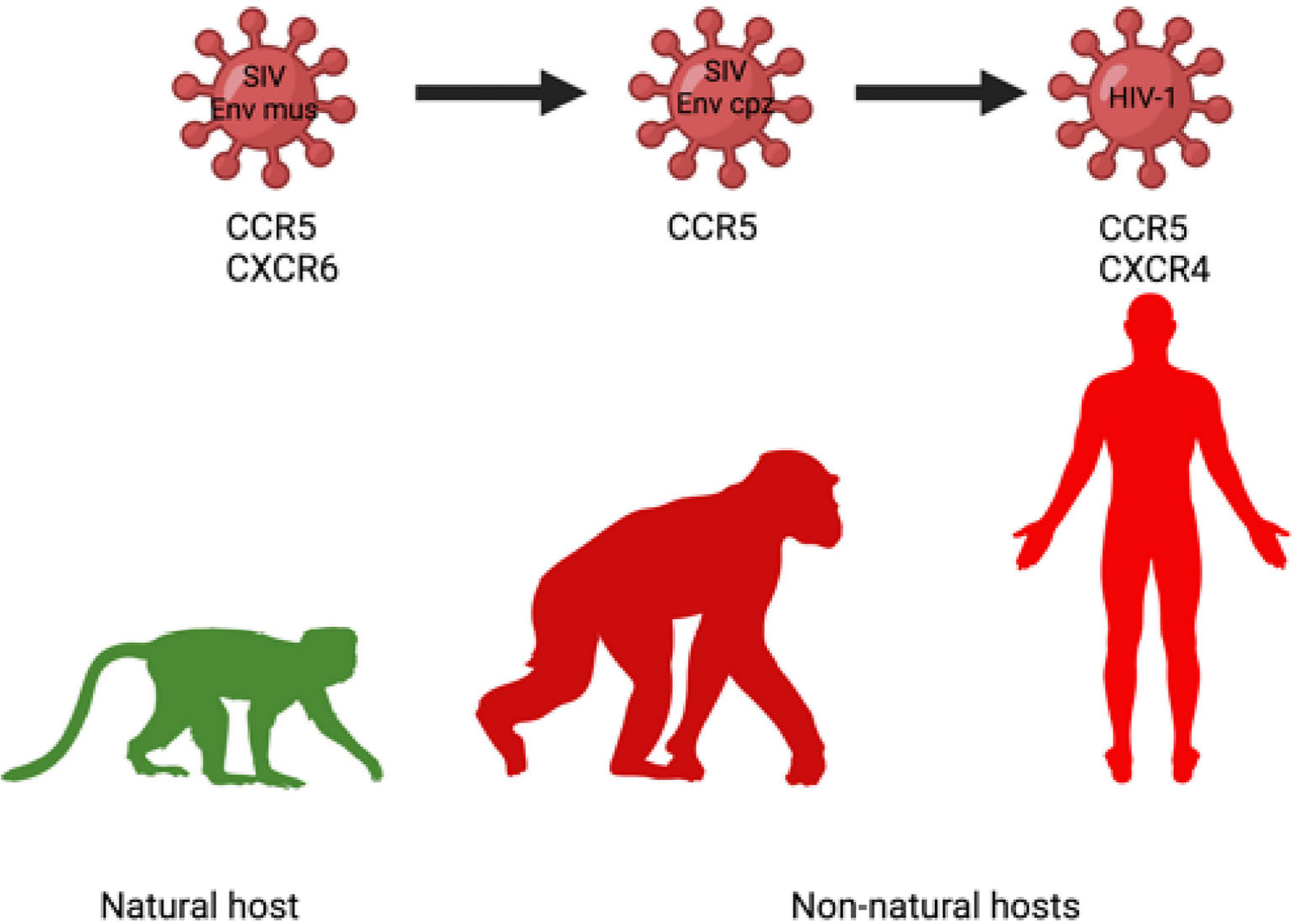
Change of coreceptor tropism (loss of CXCR6 usage) in the SIVcpz/HIV-1 lineage may contribute to increased use of CCR5 as an entry coreceptor, more widespread infection of target cells and enhanced pathogenicity of SIVcpz/HIV-1 in chimpanzee and human, respectively (187).

SIVcpz infecting chimpanzees and HIV-1 infecting humans are members of the same virus lineage and are both pathogenic (**Figure 3**). Both can use CCR5 as a principal entry coreceptor, but cannot use CXCR6, which was a coreceptor used for the entry of the ancestor of HIV-1 originated from cross-species transmission of SIVcpz infecting chimpanzees (46). In chimps, SIVcpz emerged from a *Cercopithecus* lineage of SIV (188), which *env* gene has a recombinant origin SIVgsn/mus/mon from greater spot-nosed monkey (*Cercopithecus nictitans*), mustached monkey (*C. cephus*), and mona monkey (*C. mona*) (189, 190). Contemporary SIVmus, similarly to SIVsmm and SIVagm, uses both CCR5 and CXCR6 for cell entry (187), suggesting the CXCR6 usage as a major coreceptor is attributable to SIVs with nonpathogenic course of infection in their respective hosts MUS, SM, and AGM. In the SIVcpz and HIV-1 lineage, the ability to infect *via* CXCR6 was lost and the viruses shifted their tropism exclusively towards CCR5. CXCR6 is expressed on CD4^+^ effector memory T-cells, yet on a subpopulation distinct from those with expression CCR5 (187) and therefore the switch in coreceptor use resulted in the change of target cells, probably to more vulnerable cell subsets what can lead to pathogenesis (187).

Note, however, that the main limitation of this hypothesis that SIV pathogenicity in different species is dependent on the coreceptor usage is that SIVagm and SIVsmm can actually be highly pathogenic in macaques upon direct cross-species transmission (137, 191). In macaques, both SIVsmm and SIVsab target preferentially memory cells and not naïve cells (137, 191, 192). Therefore, in order to confirm this theory, comparative pathogenesis studies in the AGMs/SMs versus macaques should be carried out to show that the viruses restrict coreceptor usage to exclusively CCR5 upon cross-species transmission.

Thus, the SIV coreceptor expansion may enable the viruses to circumvent the lowered CCR5 expression in the natural hosts, making SIV infection in natural hosts less dependent on the use of a CCR5 coreceptor (193). In more recent hosts, high and unaltered CCR5 expression appears indispensable for HIV-1 infection in humans and SIVmac infection in RMs (33–35, 84). Differential cell targeting between natural and non-natural hosts could contribute to different outcomes between infection in natural and non-natural hosts (194), but *in vivo* studies are needed to confirm these *in vitro* observations and infection of naïve cells in natural hosts of SIVs.

A more promiscuous coreceptor usage allows the virus to expand their spectrum of target cells. These new target cell subsets are less essential, with short life span, and their infection has minimal impact on immune health. This can explain why low levels of CCR5 expression on CD4^+^ T cells in natural hosts does not reduce the infection of the CD4^+^ T cells, permit high viremia, and result in infection of cells with short lifespan in natural host in comparison to non-natural host. Restricted expression of CCR5 coreceptor may thus protect essential cells from infection (in particular memory CD4^+^ T-cells) and preserve immune homeostasis. Note, however, that, in pathogenic HIV infections, high pathogenicity and disease progression is associated with an expansion in coreceptor usage rather than a more restrictive coreceptor use.

## Impact for Therapies

The impact of natural CCR5 loss-of-function mutations and the phenomenon of target cell restriction *via* downregulating cell surface CCR5 expression on preventing infection or minimizing SIV/HIV pathogenesis pointed to the central role of CCR5 in the process of natural protection against SIV/HIV. It led to development of therapeutic approaches to inactivate or block the function of CCR5 gene or protein, such as chemical or antibody-based blocking of CCR5 receptors, or generation of CCR5 cells which are deficient or downregulate CCR5.

### CCR5 Blockade - Targeting the Interaction of CCR5 With HIV

CCR5 blockers mimic the effects of naturally occurring CCR5-Δ24 mutation, at least in part, with respect to inhibiting HIV-1 utilization of cell surface CCR5 for cell entry.

Maraviroc (MVC), a nonpeptidic small molecule, causing a pharmacological blockade of CCR5 signaling, was the first CCR5 antagonist approved by FDA for the treatment of patients infected with R5-using HIV-1 virus (195). MVC blocks binding of viral envelope, gp120, to CCR5 to prevent the membrane fusion and viral entry, through allosteric inhibition (i.e. without occupying the binding site for chemokines and the HIV envelope glycoprotein gp120) and without affecting CCR5 cell surface levels or associated intracellular signaling (196–198). Maraviroc administration, originally devised for HIV treatment, may have applications beyond HIV/AIDS. Rodent models either treated with maraviroc or with CCR5 knockout showed an induced recovery after traumatic brain injury (30), however, maraviroc blockade of infant macaques only marginally impacted the rate of oral SIVmac transmission (163).

Leronlimab, a CCR5-blocking monoclonal antibody, binds to the external domains of CCR5 and through a competitive mechanism prevents HIV and SIV from binding to the CCR5 coreceptor, entering the cell and replication (199, 200). Beside the CCR5 masking from SIV/HIV, leronlimab CCR5 binding does not downregulate CCR5 expression or deplete CCR5-expressing cells (199), but prevents CCL-5-induced activation and migration of inflammatory CCR5-expressing monocytes and T lymphocytes along a chemical gradient (199). In this context, leronlimab appears a an excellent prospect for treatment of diseases, in which the CCL5-CCR5 pathway is involved in the pathogenesis. Given the role of CCR5 in immune cell migration and inflammation, CCR5 blockade with leronlimab was applied in critical COVID-19 patients, and led to the reduction of the IL-6 levels, restoration of CD4/CD8 ratio, and resolution of SARS-CoV-2 burden, thus implicating CCR5 as a potential therapeutic target for COVID-19 (201).

Antibody conjugates (ACs) comprising of an antibody carrier and small molecule CCR-5 antagonist were developed to enhance the CCR5-dependent therapies, specifically, to increase their clinical effects, reduce off-target effect and toxicity, and extend the pharmacokinetic profile of the attached molecule (202). To increase the potency of CCR5 targeted therapies, anti-CCR5 monoclonal antibodies were conjugated with a CCR5 small molecule antagonist, targeting nonoverlapping epitopes (203) and with a fusion inhibitor (204). The neutralization activity of CCR5 antagonists, such as MVC, can be further extended by formulating them with long-lived carriers - chemically programmed antibodies (cpAbs) and PEGylated derivatives. Such compounds containing MVC had significantly extended pharmacokinetic profiles (205).

### Gene-Based Therapeutic Approaches

#### CCR5-Deficient Transplants

Sustained remission of HIV infection was achieved using stem-cell transplantation from donors homozygous for *CCR5* null allele Δ32/Δ32, lacking functional expression of the CCR5 coreceptor and showing HIV resistance in two patients treated for acute myelogenous leukemia (Berlin patient) and refractory Hodgkin lymphoma (London patient) (206, 207). The Berlin patient received two rounds of total body irradiation and allogeneic hematopoietic stem-cell transplantation (allo-HSCT) and the London patient underwent one HSCT procedure.

The Berlin patient achieved long-term post treatment control of HIV (206, 208, 209) and the London Patient has been in HIV-1 remission for at least 30 months with no detectable replication-competent virus in blood, CSF, intestinal tissue, or lymphoid tissue. Both these cases potentially represent cases of HIV-1 cure (207, 210–212).

While the allogenic transplantation of CCR5 deficient cells demonstrated a feasibility of cure, finding HLA-matched donors with naturally occurring homozygous CCR5 deletions is a limiting factor of this approach. Therefore, various genomic manipulations have been attempted to disrupt CCR5 function.

Gene editing and silencing technologies have been implemented to block the CCR5 function, including (i) modifications of naturally existing anti-HIV-1 ribozymes (108), (ii) gene silencing using RNA interference to suppress CCR5 (110), (iii) programmable nucleases, such as zinc-finger transcription activator-like effector nucleases (TALENs) and (iv) clustered regularly interspaced short palindromic repeat (CRISPR)-Cas (113), and engineered zinc-finger nucleases (ZFNs) (107, 112, 213, 214).

Editing the CCR5 gene *via* CRISPR-Cas9 technology was also applied to genetically modify Mauritian cynomolgus macaque embryos as the foundation for developing a model system of SIV resistance for studying SIV/HIV disease and development of therapies. Through this technology, a disrupted gene CCR5, containing homozygous deletions in CCR5 (including a 24-bp deletion region, which does not occur spontaneously in macaques) was introduced into macaque embryos and edited cells (115).

Germline editing using CRISPR-Cas9 technology was also used to introduce a null genotype of CCR5 in human embryos from an HIV discordant couple, which is similar, yet not identical to CCR5-Δ32. Twin girls with this genetic alteration were born in 2018. While the intention behind the germline inactivation of CCR5 in the human embryo appears to be protection from HIV infection in later life, this intervention evoked questions regarding the necessity of such permanent gene inactivation, while other preventive methods are available. It also evoked discussion about readiness of germline editing technology (its safety and control of target off effect) and still limited knowledge of pleiotropic function of immune genes in health, and therefore difficulty to precisely predict the effects of the introduced alterations (215).

The pleiotropic role of CCR5, which makes this chemokine receptor a promising target for therapies of various diseases, needs to be closely studied in relation to potential undesirable effects of CCR5-targeted therapies (the receptor blockage or disruption). While they can prevent the cell-to-cell spread of HIV/SIV and reduce chronic T-cell immune activation and inflammation, the inactivation of natural CCR5 function may have various unintended consequences. For example, there is increasing evidence on the prominent role of CCR5 in the differentiation, activation and migration of the CD8^+^ T cells to the sites of inflammation (216, 217), and these processes are impaired by CCR5 deficiency or blockade (218, 219). CCR5 is also highly expressed in virus-specific CD8^+^ T cells during various viral infections, including HIV-1, suggesting a role of CCR5 in the CD8^+^ T cell responses to viral infections (216, 220–222). CCR5-expressing CD8^+^ T cells display an effector memory phenotype, age-related increase in rhesus macaques, and a marked reduction during the progression of SIV disease (223). MVC treatment reduced the *in vitro* activation of CD8^+^ T-cells from SIV-infected macaques (223). This effect could be beneficial as it may reduce disease-related chronic immune inflammation; on the other hand, it may limit the CD8^+^ T-cell responses to the virus, and potentially increase a risk of the virus latency (223). The complex role of CCR5 in immune health highlights the need for studies of the CCR5-directed therapies on CD8^+^ T cell and immune health in general.

## Conclusions

CCR5 is central to HIV pathogenesis. Targeting this receptor was successfully used as an antiretroviral therapy and could be successfully expanded to either other infections or to medical areas which are unrelated to infectious diseases. Natural hosts of SIVs adapted, over an evolutionary history of millennia, to counter SIV infection by limiting the expression of the CCR5 receptor on the target cells. Meanwhile, viruses naturally infecting natural hosts of SIVs found escape routes to counter replication restrictions due to low CCR5 expression. As such, the CCR5-SIV relation represents a perfect illustration of the red queen principle which proposes that species must constantly adapt, evolve and proliferate in order to survive in contact with the opposed species (224). “Now, here, you see, it takes all the running you can do, to keep in the same place.” (225). This remarkable ability of the SIVs and their species-specific hosts to continuously adapt calls for a careful evaluation of the cure approaches targeting CCR5 expression.

## Author Contributions

AJJ, IP, and CA designed, wrote and edited the manuscript. All authors contributed to the article and approved the submitted version.

## Funding

IP and CA are supported by grants from the National Institutes of Health/National Institute of Diabetes and Digestive and Kidney Diseases/National Heart, Lung and Blood Institute/National Institute of Allergy and Infectious Diseases: RO1 HL117715 (IP), R01 HL123096 (IP), R01 HL154862 (IP), R01 DK130481 (IP), R01 DK113919 (IP/CA), R01 DK119936 (CA), R01 DK131476 (CA), R01 AI119346 (CA). The content of this publication does not necessarily reflect the views or policies of the Department of Health and Human Services, nor does mention of trade names, commercial products, or organizations imply endorsement by the U.S. Government. The funders had no role in study design, data collection and analysis, decision to publish, or preparation of the manuscript. **Figures 2** and **3** were created with BioRender.com.

## Conflict of Interest

The authors declare that the research was conducted in the absence of any commercial or financial relationships that could be construed as a potential conflict of interest.

## Publisher’s Note

All claims expressed in this article are solely those of the authors and do not necessarily represent those of their affiliated organizations, or those of the publisher, the editors and the reviewers. Any product that may be evaluated in this article, or claim that may be made by its manufacturer, is not guaranteed or endorsed by the publisher.
